# Face-based age estimation using improved Swin Transformer with attention-based convolution

**DOI:** 10.3389/fnins.2023.1136934

**Published:** 2023-04-12

**Authors:** Chaojun Shi, Shiwei Zhao, Ke Zhang, Yibo Wang, Longping Liang

**Affiliations:** ^1^Department of Electronic and Communication Engineering, North China Electric Power University, Baoding, Hebei, China; ^2^Hebei Key Laboratory of Power Internet of Things Technology, North China Electric Power University, Baoding, Hebei, China

**Keywords:** age estimation, Swin Transformer, attention mechanism, deep learning, neural networks

## Abstract

Recently Transformer models is new direction in the computer vision field, which is based on self multihead attention mechanism. Compared with the convolutional neural network, this Transformer uses the self-attention mechanism to capture global contextual information and extract more strong features by learning the association relationship between different features, which has achieved good results in many vision tasks. In face-based age estimation, some facial patches that contain rich age-specific information are critical in the age estimation task. The present study proposed an attention-based convolution (ABC) age estimation framework, called improved Swin Transformer with ABC, in which two separate regions were implemented, namely ABC and Swin Transformer. ABC extracted facial patches containing rich age-specific information using a shallow convolutional network and a multiheaded attention mechanism. Subsequently, the features obtained by ABC were spliced with the flattened image in the Swin Transformer, which were then input to the Swin Transformer to predict the age of the image. The ABC framework spliced the important regions that contained rich age-specific information into the original image, which could fully mobilize the long-dependency of the Swin Transformer, that is, extracting stronger features by learning the dependency relationship between different features. ABC also introduced loss of diversity to guide the training of self-attention mechanism, reducing overlap between patches so that the diverse and important patches were discovered. Through extensive experiments, this study showed that the proposed framework outperformed several state-of-the-art methods on age estimation benchmark datasets.

## 1. Introduction

A large amount of useful information in facial images, such as age, gender, identity, race, emotion, and so forth (Angulu et al., 2018), and research on techniques related to facial image analysis has become the focus of computer vision. What is the significance of the facial age as an important feature? As an important physical and social characteristic of human beings, age plays a fundamental role in human social interaction. Recently, age estimation based on facial images is already an important research topic (Zhao et al., 2020; Agbo-Ajala and Viriri, 2021), which predicts the age corresponding to the image containing the face in the image. The task has very good application prospects in various intelligent fields, such as cross-age face recognition, intelligent security surveillance, harmonious human-computer interaction, image and video retrieval, face-based age prediction, and marketing analysis (Bruyer and Scailquin, 1994; Geronimo et al., 2009; Song et al., 2011; Angulu et al., 2018; Pei et al., 2019).

Modern face-based age estimation methods typically consist of two directions. One is to improve the learning ability of the neural network, and the other is to use other features related to the age of the face to assist the learning of the network. Convolutional networks can learn age features in facial images by multilayer convolution and have achieved great success in the field of computer vision with a wide range of applications. With the growing popularity of convolutional neural networks (CNNs), recent work on face-based age estimation has used these networks as a backbone (Shen et al., 2018; Dagher and Barbara, 2021; Sharma et al., 2022; Zhang and Bao, 2022). Most of these works improve the learning ability of the network by increasing the number of layers of convolutional layers (Dornaika et al., 2020; Yi, 2022) and improving the structure of the network. However, as convolutional networks continue to improve, the potential of CNN-based facial age estimation models has been exploited, and the increasing number of network model parameters has raised the cost of training. Therefore, we proposed to use a new network model designed specifically for face-based age estimation. Some other recent studies on face-based age estimation (Deng et al., 2021; Lu et al., 2022; Wang et al., 2022) have improved the accuracy of age estimation by extracting age features of faces and the relationship between different ages (Akbari et al., 2020; Xia et al., 2020). These studies have enhanced the learning ability of the network using attention-related mechanisms, using features other than faces such as sex, gender (Liu et al., 2020), and label distribution (Zeng et al., 2020; Zhao et al., 2020). However, these efforts may destroy the features and the structure of the original image while extracting the age features of the images, resulting in the loss of age information. Therefore, how to extract features without destroying the extracted features and the structure of the original image is a research direction.

Recently, the self-attention mechanism and Transformer model (He et al., 2021) have attracted great attention in computer vision and natural language processing tasks. Vision Transformer (Dosovitskiy et al., 2020) has shown that the Transformer-based model indeed contains the capacity as the backbone network instead of former pure CNN model in image synthesis and classification tasks (He et al., 2016; Szegedy et al., 2017). Related research (Bourdev et al., 2011) shows that compared with CNN, the self-attention mechanism of Transformer is not limited by local interactions and allows both long-distance dependencies and computes in parallel and learn the most appropriate inductive bias according to different task goals, which has achieved good effects in many vision tasks. The attention mechanism (Niu et al., 2021) is also a direction to improve the prediction ability of the network, and the self-attention mechanism (Lin et al., 2017) of the Transformer can quickly extract the important features of sparse data, which is an improvement of the attention mechanism. It reduces the reliance of the network on external information and is better at capturing the correlation within the data or features. Therefore, the self-attention mechanism can be designed to exploit age-specific patches during training to boost the performance of face-based age estimation methods.

**FIGURE 1:**
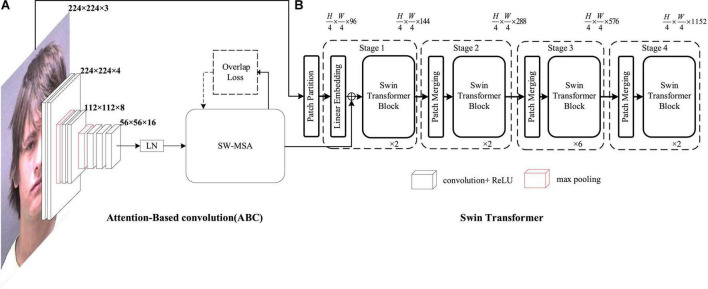
Structure of an improved Swin Transformer with attention-based convolution (ABC). **(A)** Structure of an attention based convolution. **(B)** Swin Transformer.

In this study, we proposed an attention-based convolution (ABC) age estimation framework called an improved Swin Transformer with ABC. The architecture of an improved Swin Transformer with ABC is illustrated in Figure 1. The core of the improved Swin Transformer with ABC was the ABC. In ABC, two separate regions were implemented, namely shallow convolution and a multiheaded self-attention mechanism. The feature extractor performed initial feature extraction of the image using a shallow convolutional network and then further extracted the corresponding patches that might contain rich age-specific information through the multiheaded attention mechanism, according to the number of probes. ABC extracted facial patches containing rich age-specific information by a shallow convolutional network and multiheaded attention mechanism. Subsequently, the features obtained by ABC were spliced with the flattened image in the Swin Transformer, which were then input to the Swin Transformer to predict the age of the image. ABC also introduced loss of diversity to guide the training of self-attention mechanism, reducing overlap between patches so that the diverse and important patches were discovered. The contributions of this study were as follows:

(1) We proposed a new module called ABC that used shallow convolution and shifted window self-attention mechanism. The image was initially extracted and refined by shallow image convolution, and some facial regions containing rich age-specific information were extracted by the multiheaded self-attention mechanism.
(2) We introduced the Swin Transformer as the backbone model, and the features obtained by ABC were spliced with the output of the embedding layer of the Swin Transformer in the channel dimension and subsequently input to the rest of the Swin Transformer so that the model could better capture the global and local information of the image and achieve better results by learning the relationship between different features of the image.
(3) We introduced a new loss function that made each probe of the multiheaded self-attention mechanism reveal a different age region and prevent overlapping feature extraction from the multiheaded feature extractor.

## 2. Related research

We reviewed and discussed related studies on face-based age estimation to lay the foundation for this study. We also reviewed the attention mechanism and Transformer, both of which were relevant to the proposed ABC.

### 2.1. Face-based age estimation

In the last few decades, many researches have been conducted on face-based age estimation. One of the earliest studies can be traced back to (Kwon and da Vitoria Lobo, 1999), in which the researcher’s classified faces into three age groups based on the craniofacial development theory and wrinkle analysis. In the traditional face-based age estimation methods, first the face-based age features are extracted, and then classification and regression models are established for face-based age estimation. With the rapid development of deep learning in recent years, deep learning–based facial age estimation methods have significantly improved the accuracy and robustness of face-based age estimation, especially the accuracy of face-based age estimation under unconstrained conditions.

Yi et al. (2014) proposed a multistream CNN to better leverage high-dimensional structured information in facial images. The authors cropped multiple patches from facial images so that each stream learned from one patch. Then, the features extracted from different patches were fused before the output layer. Ranjan et al. (2015) used a deep convolutional neural network (Chen et al., 2016) with 10 convolutional layers, 5 pooling layers, and 1 fully connected layer to extract the age characteristics of facial images. Then, age regression was performed using a three-layer artificial neural network. One of the first studies to use CNNs for the face-based age estimation problem was (Wang et al., 2015), in which a CNN with two convolutional layers was deployed. Han et al. (2017) used a modified AlexNet (Krizhevsky et al., 2017) to develop a multitask learning method for heterogeneous face attribute estimation including the age. Rothe et al. (2018) transformed the regression problem into a classification-regression problem and proposed a deep expectation network (DEX) for the age estimation of representations. The DEX network changed the number of neurons in the last layer of the VGG-16 network.

In general, CNN-based methods for face-based age estimation can be divided into two categories. The first approach is to use deeper and better deep learning models as backbone networks with better networks to extract features (Dornaika et al., 2020; Lu et al., 2022; Sunitha et al., 2022; Wang et al., 2022). The second approach is to improve the performance of the network in terms of other attributes of the face, such as race features and gender features (Zhang et al., 2017a; Liu et al., 2020; Deng et al., 2021), and relational features of different ages (Song et al., 2011; Gao et al., 2017; Zeng et al., 2020). In our previous study, we proposed a multilevel residual network model to further improve the performance of the network so as to better improve the accuracy of age estimation (Levi and Hassner, 2015). As the CNN-based network model for face-based age estimation may soon reach a bottleneck in the direction of face-based age estimation, we tried to incorporate a newer network model that combined the advantages of CNN with the advantages of the new network to achieve better results.

### 2.2. Attention-based facial age estimation

Attention mechanism (Vaswani et al., 2017) mimics the internal processes of biological observation behavior, increasing the fineness of observation in certain regions. The attention mechanism can quickly extract important features of sparse data and is therefore widely used in machine translation, speech recognition, and image processing (Wang et al., 2016), and other fields. A multiheaded attention mechanism can attend to multiple informative segments of the input with an attention head attending to one specific segment. Therefore, the number of segments that the multiheaded attention mechanism can attend to is determined by the number of attention heads. In computer vision, the self-attention layer takes the feature map as input and calculates the attention weights between each pair of features to generate an updated feature map where each position has information about any other feature in the same image. These layers can replace convolution directly or be combined with convolution layers. They are able to handle larger perceptual fields than conventional convolution, and therefore can acquire dependencies between some long-distance interval features on the space.

Multiple attention mechanisms have been proposed for visual tasks to address the weaknesses of convolutions due to the aforementioned characteristics. Bello et al. (2019) proposed to augment convolutional networks with self-attention by concatenating convolutional feature maps with a set of feature maps produced *via* a novel relative self-attention mechanism. Hu et al. (2018) proposed a simple, lightweight approach for better context exploitation in CNNs by introducing a pair of operators: gather (which efficiently aggregated feature responses from a large spatial extent) and excite (which redistributed the pooled information to local features). Chen et al. (2018) proposed the “double attention block,” which was designed with a double attention mechanism in two steps, a novel component that aggregated and propagated informative global features from the entire spatiotemporal space of input images/videos, enabling subsequent convolution layers to access features from the entire space efficiently. The nonlocal operation proposed by Wang et al. (2018) computed the response at a position as a weighted sum of the features at all positions and achieved excellent results in the experiment. Wang et al. (2022) proposed a face-based age estimation framework called attention-based dynamic patch fusion (ADPF). The ADPF dynamically located and ranked age-specific patches by employing a novel ranking-guided multihead hybrid attention mechanism and used the discovered patches along with the facial image to predict the age of the subject.

A previously proposed work (Bello et al., 2019) adds an attention mechanism to the convolutional network to enhance the convolutional network. A previously proposed work (Hu et al., 2018) enhances the input image by using the attention mechanism to get the feature responses of adjacent regions of the image. A previously proposed work (Chen et al., 2018) get the global features and local features by two attention mechanisms, respectively. The previously proposed work (Wang et al., 2018, 2022) learn the important features by calculating the region weights through the attention mechanism. Different from the above work which uses the attention mechanism to enhance the deep convolutional network, our work uses shallow convolution and attention mechanism with the aim of finding the important regions without destroying the original image and stitching these regions with the original image to enhance the Transformer network.

### 2.3. Vision transformer

**FIGURE 2:**
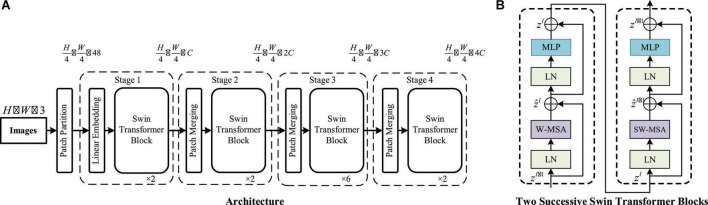
Architecture of a Swin Transformer: **(A)** Structure of a Swin Transformer (Swin-T). **(B)** Two successive Swin Transformer blocks. Window based self-attention (W-MSA) and shifted window based self-attention (SW-MSA) are multihead self-attention modules with regular and shifted windowing configurations, respectively (Vaswani et al., 2017).

Transformer (Vaswani et al., 2017) is a kind of deep divine meridian based on the self-attention mechanism. In recent years, Transformer-based models have become a popular research direction in the field of computer vision. Another visual Transformer model, ViT, recently proposed by Dosovitskiy et al. (2020), achieved state-of-the-art performance on several image recognition benchmark tasks in a structure that fully adopted the standard structure of a Transformer, ViT. Liu Z. et al. (2021) proposed the Swin Transformer, which enabled the flexibility of the Transformer model to handle images of different scales by applying a hierarchical structure similar to that of CNN. The Swin Transformer used a windowed attention mechanism to greatly reduce the computational complexity. The architecture of a Swin Transformer is illustrated in Figure 2. Yuan et al. (2021) proposed CeiT, combined with the ability of CNN to extract low-level features, to design an Image-to-Tokens module, which extracted the patch from the generated low-level features. Wang et al. (2021) proposed the CrossFormer, which used a cross-scale embedding layer (CEL), generated patch embeddings using a CEL at each stage, and extracted features using four different-sized convolutional kernels in the first CEL layer. The features were extracted using four convolutional kernels of different sizes, and the results of the convolutional kernels were stitched into patch embeddings. Peng et al. (2021) proposed Conformer (Yuan et al., 2021)], which combined the features of a Transformer and CNN through a parallel structure to achieve feature fusion by bridging each other, so that the local features of CNN and the global features of the Transformer could be retained to the maximum extent. Xiao et al. (2021) proposed ViTc, which replaced the patch embedding module in the Transformer with convolution. It made the replaced Transformer more stable and converge faster. Liu Z. et al. (2021) proposed the TransCNN by introducing a CNN layer after the self-attention block so that the network could inherit the advantages of a Transformer and CNN. UniFormer (Li et al., 2022) seamlessly integrated the advantages of convolution and self-attention through the Transformer to aggregate the local and global features at shallow and deep layers, respectively, solving the problem of redundancy and dependency for efficient representation learning.

A previous study proposed (Yuan et al., 2021) replacing the original three structures of the Transformer with convolutional layers in the Transformer, thus integrating CNN into the Transformer. Another previous study proposed (Wang et al., 2021) designing a CEL and long short distance attention to replace the Transformer block and MSA (Multihead Self-Attention) in the Transformer. A related study (Li et al., 2022) seamlessly integrated the merits of convolution and self-attention in a concise Transformer format and proposed a new framework UniFormer. Some previous studies proposed (Wang et al., 2021; Yuan et al., 2021; Li et al., 2022) combining CNN with the structure in the Transformer block to improve the capabilities of the network by adding or replacing it so as to improve the framework of the Transformer. However, the present study designed an independent shallow convolution, which extracted features through a self-attentive mechanism to be stitched with the original image and input to the Transformer, without modifying the Transformer. The CNN performed the role of extracting shallow features in the network while preserving the original structural information of the image. Another study (Xiao et al., 2021) introduced convolution to process the input original image by replacing the patch layer with CNN. A related study (Liu Z. et al., 2021) viewed image patches as tokens by CNN, which continuously extracted features through a multilayer self-attention mechanism and finally input to the Transformer block. The present study extracted shallow features by shallow convolution without destroying the location structure information of the image. The feature regions rich in face-based age information were obtained through a self-attention mechanism and stitched with the original image after the patch layer processing in the Transformer, instead of directly processing the original image. A related study (Peng et al., 2021) combined the features of the Transformer and CNN through a parallel structure using a bridge to achieve feature fusion. This study introduced CNN and self-attention mechanism to extract shallow features while preserving the original structural information of the image. The purpose of the CNN and self-attention mechanism was to improve the ability of the Transformer to obtain feature dependencies at long distances.

## 3. Methodology

In this section, we first discussed the core of the Swin Transformer with the attention-based convolution mechanism, which was the proposed ABC. Then, we combined the ABC and Swin Transformer parts. Finally, we introduced the age expectation and loss function. The architecture of the Swin Transformer with the ABC mechanism is shown in Figure 1.

### 3.1. ABC

**FIGURE 3:**
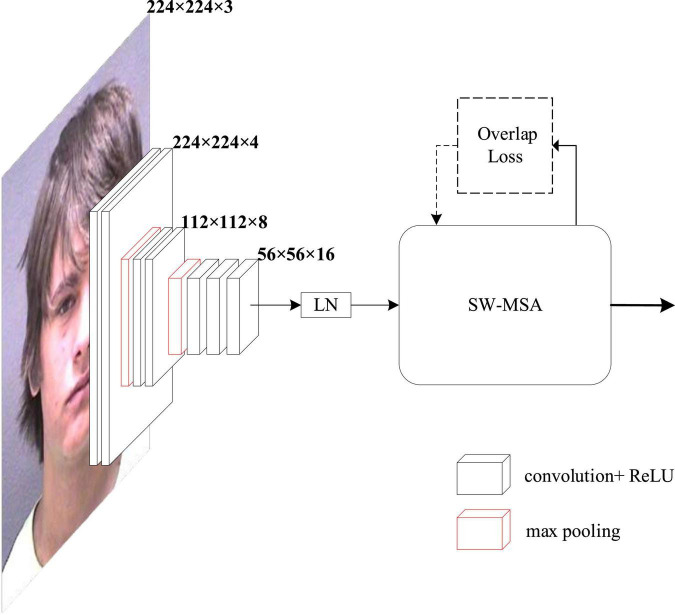
Structure of the proposed attention-based convolution (ABC).

As the Swin Transformer with attention-based convolution is based on ABC and the critical component of ABC is the self-attention mechanism, we first introduced the self-attention mechanism followed by the proposed attention-based convolution. Finally, we detailed the complete mechanism. The architecture of attention-based convolution is shown in Figure 3.

We considered an input tensor *X* with a dimension of *h* × *w* × *c*, where *h* denotes the height, *w* denotes the width, and *c* denotes the number of channels. During training, the input to ABC was a fixed-size 224 × 224 RGB image.

This model started with the institutional area of the convolution. The image was passed through a stack of convolutional layers; this convolutional region had eight convolutional layers, where we used filters with a very small receptive field: 3 × 3. The convolution stride was fixed to 1 pixel; the spatial padding of the convolution layer input was such that the spatial resolution was preserved after convolution, that is, the padding was one pixel for 3 × 3 convolution layers. Spatial pooling was carried out by two max-pooling layers, which followed the second and fourth convolutional layers. Max-pooling was performed over a 2 × 2 pixel window, with stride two. All hidden layers were equipped with the rectification nonlinearity.

The convolution was followed by the region of the self-attention structure. The output of the convolutional layer *X* was used as the input of the attention mechanism. Let us consider an input tensor *X* with a dimension of *h* × *w* × *c*, where *h* denotes the height, *w* denotes the width, and *c* denotes the number of channels. *X* was convolved into three separate tensors: *Q* with a shape of *h* × *w* × *c*_*Q*_, *K* with a shape of *h* × *w* × *c*_*K*_, and *V* with a shape of *h* × *w* × *c*_*V*_, where *c*_*Q*_, *c*_*k*_, and *c*_*V*_ indicate the number of channels in the corresponding tensor. The intention behind self-attention was to compute a weighted summation of the values, *V*, where the weights were computed as the similarities between the query, *Q*, and the corresponding key, *K*. Therefore, to compute the similarity, *Q* and *K* normally had the same shape, that is, *c*_*Q*_ = *c*_*V*_. The output of a single self-attention mechanism was computed as:


(1) $$S_{n_{1}}=s\,o\,f\,t\,\max\left(\frac{Q^{\prime }\cdot K^{\prime ,T}}{\sqrt{c_{K}}}\right)\cdot V$$

where *Q*′ and *K*′ are flattened tensors to perform the dot product.

After the scaling operation, that is, dividing the similarity matrix *Q*′⋅*K*′^*T*^ by a factor of $\sqrt{c_{K}}$ and applying the softmax function, we performed a dot product between the normalized similarity matrix and *V* to generate the self-attention maps *S*_*n*_ with a dimension of *h* × *w* × *c*_*K*_. *n*_*i*_ is the number of heads of attention probes in the multiheaded attention mechanism.

As we flattened the two-dimensional feature maps into a one-dimensional vector in Equation 1, the original structure of the feature maps was distorted. We adopted the relative positional encoding to make it efficient when dealing with structured data such as images and multidimensional features. Specifically, the relative positional encoding was represented by the attention logit, which encoded how much an entry in *Q*′ attended to an entry in *K*′. The attention logit was computed as:


(2) $$l_{i,j}=\frac{q_{i}^{T}}{\sqrt{c_{K}}}\,\left(k_{j}+r_{j_{x}-i_{x}}^{w}+r_{j_{y}-i_{y}}^{h}\right)$$

where *q*_*i*_ is the *i*th row in *Q*′ indicating the feature vector for pixel *i*:=(*i*_*x*_,*i*_*y*_) and *k*_*j*_ is the *j*th row in *K*′ indicating the feature vector for pixel *j*:=(*j*_*x*_,*j*_*y*_). $r_{j_{x}-i_{x}}^{w}$ and $r_{j_{y}-i_{y}}^{h}$ are learnable parameters encoding the positional information within the relative width *j*_*x*_−*i*_*x*_ and relative height *j*_*y*_−*i*_*y*_, respectively. With the relative positional encoding, the output of a single self-attention mechanism could be reformulated as:


(3) $$S_{n_{1}}=s\,o\,f\,t\,\max\left(\frac{Q^{\prime }\cdot K^{\prime ,T}+m_{h}+m_{w}}{\sqrt{c_{K}}}\right)\cdot V$$

where $m_{h}\,\left[i,j\right]\,=\,q_{i}^{T}\,r_{j_{y}-i_{y}}^{h}$ and $m_{h}\,\left[i,j\right]\,=\,q_{i}^{T}\,r_{j_{x}-i_{x}}^{w}$ are matrices of relative positional logits. In this study, the number of heads of the attention probe in the multiheaded attention mechanism was set to four.

A key design element of ABC was its shift of the window partition between consecutive self-attention layers, as illustrated in Figure 2. The shifted windows bridged the windows of the preceding layer, providing connections among them that significantly enhanced the modeling power. As illustrated in Figure 2, the module used a regular window partitioning strategy that started from the top-left pixel, and the 56 × 56 feature map was evenly partitioned into 8 × 8 windows of size 7 × 7 (*M* = 7). Then, the module adopted a windowing configuration that was shifted from that of the preceding layer by displacing the windows by $\left(\left[\frac{M}{2}\right],\left[\frac{M}{2}\right]\right)$ pixels from the regularly partitioned windows.

With the shifted window partitioning approach, the attention blocks were computed as follows:


(4) $${\hat{X}}_{1}=W-M\,S\,A\,\left(\hat{X}\right)$$

where ${\hat{X}}_{1}$ denotes the output features of the shallow convolution module.

### 3.2. Swin Transformer with ABC mechanism

We again used the sample image input at the beginning with the tensor *Y* as input to the Transformer. First, the tensor *Y* went through the patch partition layer and the dimension became 56× 56× 48. Then, *Y* was again mapped to the specified dimension by the linear embedding layer, and the dimension of *Y* was 56× 56× 128. The role of the patch partition module was to crop a patch_size * patch_size block of the input original image by conv2d. The patch_size was set to four. The specific structure of the Swin Transformer is shown in the Figure 2. The remaining structure of the Swin Transformer could be referred from the original study, and as no modifications were made to the Swin Transformer in this study, it was not repeated in this study.

In the output of the self-attention mechanism in Equation 4, which was the output of ABC, the output tensor *X* had a dimension of 56× 56× 16 and the output of the linear embedding layer of the Swin Transformer also had a dimension of 56× 56× 128, so we considered fusing the two tensors. We spliced the output *X* of ABC with the output *Y* of the embedding layer in the channel dimension to get *Y*_1_, that had a dimension of 56× 56× 144. Then, we replaced *Y* with the spliced tensor *Y*_1_, continued with the network layer behind the Swin Transformer, and finally got the final output *Z* at the end of the Swin Transformer.

Using a Transformer as the backbone network can make the model better to capture the global and local information of images, extract more powerful features, and achieve better effects by learning the relationship between different features of images. This specific framework can more effectively learn the long-distance dependencies in face semantic parts, which naturally helps model the strong attribute correlations (Liu et al., 2020).

Focusing on the ability of the Swin Transformer to mine both long-range dependencies and parallel computation, we first obtained several important age feature regions rich with age-specific information using ABC and stitched them with the original input image. This way, the Swin Transformer afterward could learn more dependencies between important age features and improve the learning ability of the network. We used the stitching in the connection part of the ABC and Swin Transformer. By stitching in the channel dimension, the stitched *Y*_1_ at this point not only retained the structure and features of the original input image but also had feature regions that were processed by the shallow convolutional layer and the multiheaded attention mechanism. The ABC-derived feature regions not only highlighted the regions containing rich age information but also retained the structural information of the regions to the greatest extent by the shallow convolutional layer. The Swin Transformer could learn the relationship between the features of the original input image and the features obtained by ABC when processing the stitched *Y*_1_, thus fully exploiting the advantage of the Swin Transformer. The use of the ABC+Swin Transformer enabled the model to better capture the global and local information of the image, which not only preserved the local sensitivity and translation invariance of CNN but also improved the ability of Swin Transformer to learn the dependencies between features for better results.

### 3.3. Loss

We used the label distribution learning to learn the exact age and Kullback–Leibler divergence to learn the age distribution to estimate the age.

Formally, let *x*_*i*_ ∈ *X* denote the *i*th input instance with *i* = 1,2,…,*N*, and ${\hat{y}}_{i,j}$ denote the softmax distribution of the predicted values of the network, where *N*is the number of instances and *c* is the number of each age.

Deep label distributions transform *y*_*i*_ from a single class label to a label distribution and then predict ${\hat{y}}_{i}$ by label distribution learning. Instances with the same class label *y*_*i*_ share the identical Gaussian distribution:


(5) $$l_{x_{i}}^{c_{j}}=\frac{1}{\sqrt{2\,\pi }\,\sigma \,M}\,\exp\left(-\frac{{\left(c_{j}-y_{i}\right)}^{2}}{2\,\sigma^{2}}\right)$$

where $l_{i}^{c_{j}}$ is the degree to which the value of each age *c*_*j*_ describes images *x*_*i*_ and *c*_*j*_ = 0,1,…,*C*; σ is the standard deviation of $l_{b}^{c_{i}}$; and the factor *M* lead $\sum_{j=0}^{a}l_{x_{i}}^{c_{j}}=1$. In this study, σ was set to 2.

KL (Kullback-Leibler) tried to generate the predicted softmax probability distribution as similar to the ground truth distribution as possible. For the same random variable, with two different distributions ${\hat{y}}_{i}$ and $l_{i}^{c_{j}}$, the *KL* scatter of ${\hat{y}}_{i}$ to $l_{i}^{c_{j}}$ was expressed as:


(6) $$K\,L=\sum_{x}{\hat{y}}_{i}\times \log_{2}\frac{{\hat{y}}_{i}}{l_{i}^{c_{j}}}$$

The less the result of KL_*loss*_, the less the difference between the two divisions, indicating that the less the difference between the true age and the estimated age, the more accurate the age estimated by the network.

The number of patches that could be discovered was determined by the number of attention heads implemented in ABC. However, during implementation, we found that patches tended to overlap, especially in informative regions. This overlap of attended patches might lead to redundant learning sources and leave other age-specific patches undiscovered. To alleviate this overlap issue, we used a diversity loss to learn diverse and nonoverlapping patches by minimizing the summation of products of corresponding entries in two attention heads, *S_n_*_1_(*h*′,*w*′) and *S_n_*_2_(*h*′,*w*′). The diversity loss was formulated as:


(7) $$L_{O\,v\,e\,r\,l\,a\,p}=\sum_{\begin{array}{c} n_{1},n_{2}\\ n_{1}\neq n_{2} \end{array}}^{n}\sum_{h^{\prime }}^{h}\sum_{w^{\prime }}^{w}S_{n_{1}}\,\left(h^{\prime },w^{\prime }\right)\cdot S_{n_{2}}\,\left(h^{\prime },w^{\prime }\right)$$

Each probe in the multiheaded attention mechanism generated a *S*_*n*_(*h*′,*w*′), and *S*_*n*_(*h*′,*w*′) represented the region that the probe focused on. *S*_*n*_(*h*′,*w*′) could be regarded as a weight matrix with dimension 56× 56× 16. In *S*_*n*_(*h*′,*w*′), the richer the region with age-specific information, the larger the weight matrix corresponding to that region. When the result obtained by multiplying *S*_*n*_(*h*′,*w*′) and the other *S*_*n*_(*h*′,*w*′) was 0, the regions attended by the two attention probes did not overlap. When the overlap loss obtained after multiplying all *S*_*n*_(*h*′,*w*′) with each other was zero, no overlap occurred between the regions attended by different probes, which prevented the redundancy of learning caused by multiple attention probes attending to the same region at the same time.

The overall loss to train this network was the summation of the two loss functions:


(8) $$L=L_{K\,L}+\lambda_{1}\,L_{O\,v\,e\,r\,l\,a\,p}$$

where λ_1_ is the hyperparameter that attempts to balance the influences of mean and residual sublosses in the combined loss function. In this study, λ_1_ was set to 10.

The estimated age of the *i*-th test image could be calculated based on Equation 9. Each descriptive degree in the label distribution was multiplied by its corresponding label and then summed to obtain the final predicted age. Assuming that the network softmax layer output 62 probability values from age 16 years to age 77 years, *p*_*i*,*j*_ corresponded to the probability of the prediction for 16–77 years, respectively, as shown in Equation 9:


(9) $${\hat{y}}_{i}=\sum_{j=16}^{77}{\hat{y}}_{i,j}\times c_{j}$$

## 4. Experiments

In this section, we first detailed the experimental settings and then compared our method with state-of-the-art studies on face-based age database MORPH Album II (Ricanek and Tesafaye, 2006), FG-NET dataset (Cootes et al., 2001), and Adience dataset (Eidinger et al., 2014). We removed the important design elements of ABC. In this study, Swin Transformer used the model parameter settings of the base.

### 4.1. Implementation details

The effectiveness of the methods in this study was demonstrated using the model as the backbone network for image classification tasks. For training and testing on the MORPH Album II, FG-NET, and Adience datasets, the size was set to 64, the number of iterations to 800, the weight decay to 0.005, the momentum to 0.9, and the default learning rate to 0.0001. The learning rate was multiplied by 0.1 when training to the 500th iteration, and by 0.01 when training to the 650th iteration.

The facial images in MORPH Album II and FG-NET datasets had corresponding specific age values. For the evaluation metrics of these two datasets, we used MAE. The age labels corresponding to the images in the Adience dataset were age groups, such as 0–2 and 4–6. For the evaluation metrics of the Adience dataset, we used the accuracy of a one-class classification.

The MAE was calculated as shown in Equation 10, where *y*_*j*_ is the age label value, $y_{j}^{\prime }$ is the age prediction value, and *N* is the number of test images. The accuracy of the one-class classification was calculated as shown in Equation 11, where *TP* is the correctly predicted sample and *FP* is the incorrectly predicted sample.


(10) $$\text{MAE}=\frac{1}{N}\,\sum_{j=1}^{N}\left|y_{j}-y_{j}^{\prime }\right|$$

(11) $$p\,r\,e\,c\,i\,s\,i\,o\,n=\frac{T\,P}{T\,P+F\,P}$$

### 4.2. Dataset

We conducted experiments on three commonly used face-based age estimation benchmark datasets: MORPH Album II, FG-NET, and Adience.

**FIGURE 4:**
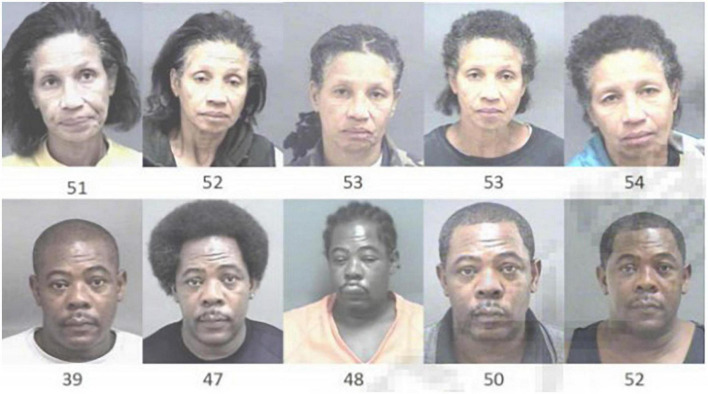
Images in the MORPH Album II dataset.

The MORPH Album II is one of the most common and largest longitudinal face databases in the public domain for age estimation, containing 55,134 facial images of 13,617 subjects. The age ranged from 16 to 77 years with an average age of 33 years, and the male-to-female ratio was 5.5:1. Each facial image in the MORPH II dataset was associated with identity, age, race, and sex labels. We randomly split the whole dataset into two subsets: one with 80% of the data for training and the other with 20% for testing. In this setting, no identity overlap occurred between the two subsets. Some images are shown in Figure 4.

**FIGURE 5:**
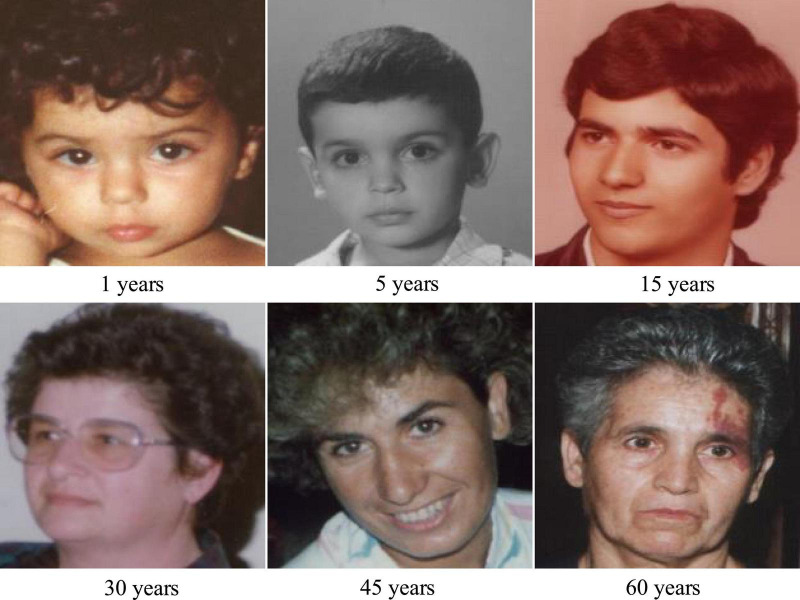
Images in the FG-NET dataset.

The FG-NET dataset contained 1,002 facial images from 82 noncelebrity subjects with large variations in lighting, pose, and expression. The age ranged from 0 to 69 years (on average, 12 images per subject). Each subject in this dataset had more than 10 facial images taken over a long time span. In addition, the facial images in this dataset contained pose, illumination, and expression variations. For the FG-NET dataset, we use the leave-one-person-out (LOPO) strategy. In each fold, we use facial images of one subject for testing and the remaining images for training. Since there are 82 subjects, this process consists of 82-fold and the reported results are the average values. Some images are shown in Figure 5.

**FIGURE 6:**
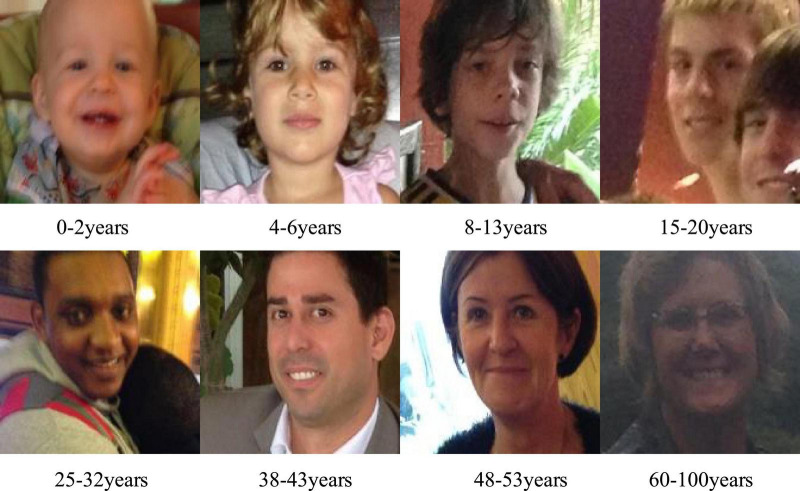
Images in the Adience dataset.

The Adience dataset was collected from the photos taken by users themselves from the Yahoo image-sharing website. It contained 26,580 images of 2,284 people with age ranging from 0 to 100 years. The labels used in this dataset were age group labels: 0–2, 4–6, 8–13, 15–20, 25–32, 38–43, 48–53, and 60–100, categorized into eight groups. Some images with age labels were labeled as age values, some as other age ranges, and some others as empty. In this study, the images with age labels labeled as age values, other age ranges, and null were removed. The age classification experiments using the Adience dataset were treated as eight classes. Some images are shown in Figure 6.

### 4.3. Evaluations on the MORPH Album II dataset

The MAE values for the aforementioned settings of the MORPH Album II dataset are tabulated in Table 1. As shown in the table, the ABC+Swin Transformer outperformed all state-of-the-art methods. Compared with the other methods on the MORPH Album II, our proposed method improved by 55.45% over the LDL method, by 37.2% over the VGG method, by 15.6% over the VADL method, and by 14.6% over the ADPF method. Compared with the original Swin Transformer, our proposed ABC+Swin Transformer improved by 8.4%. The experimental results showed that our method had high accuracy on the MORPH Album II dataset and improved the learning ability of the facial image age of the Swin Transformer.

**TABLE 1:** Comparison of age classification on the MORPH Album II dataset.

Method	MAE
OHRank (Chang et al., 2011)	8.83
MTWGP (Zhang and Yeung, 2010)	6.28
OHRank (Chang et al., 2011)	6.07
CA-SVR (Chen et al., 2013)	5.88
SVR (Guo et al., 2008)	5.77
LDL (Geng et al., 2013)	4.87
DLA (Wang et al., 2015)	4.77
ALDL (Geng et al., 2013)	4.34
KPLS (Guo and Mu, 2011)	4.18
BIF+KCCA (Guo and Mu, 2013)	3.98
VGG (Rothe et al., 2016)	3.45
ARN (Agustsson et al., 2017)	3.25
VGGNetHybrid (Xing et al., 2017)	2.96
DAG-VGG16 (Li et al., 2019)	2.81
Mean-Variance Loss (Pan et al., 2018)	2.80
MSFCL-KL (Xia et al., 2020)	2.73
DEX (IMDB-WIKI) (Rothe et al., 2018)	2.68
VDAL (Liu H. et al., 2020)	2.57
ADPF (Wang et al., 2022)	2.54
Anet+Gnet+Rnet (Deng et al., 2021)	2.47
Swin Transformer (Liu Z. et al., 2021)	2.37
ABC+Swin Transformer (Ours)	**2.17**

Bold values represent the best data.

### 4.4. Evaluations on the FG-NET dataset

As the number of images in the FG-NET dataset was too small, training the dataset directly might lead to a decrease in the accuracy of the results and slow convergence. Therefore, we used the pretraining weights obtained in the aforementioned FG-NET dataset as the initial weights for training. The MAE values for the aforementioned settings of the FG-NET dataset are tabulated in Table 2. As shown in the table, the ABC+Swin Transformer outperformed all state-of-the-art methods. Compared with the other methods on the FG-NET dataset, the method proposed in this study improved by 21.8% over the DAG-VGG16 method, by 15.8% over the ADPF method, and by 8.6% over the BridgeNet method. Compared with the original Swin Transformer, the performance of our proposed ABC+Swin Transformer improved by 4.8%. The experimental results showed that our method had high accuracy on the FG-NET dataset and improved the learning ability of facial image age of the Swin Transformer.

**TABLE 2:** Comparison of age estimates on the FG-NET dataset.

Method	MAE
Mean-Variance Loss (Pan et al., 2018)	4.10
DRFs (Dagher and Barbara, 2021)	3.85
M-LSDML (Liu et al., 2017)	3.74
DLDFL (Shen et al., 2019)	3.71
DFR (Shen et al., 2019)	3.47
DAG-VGG16 (Taheri and Toygar, 2019)	3.08
DAG-GoogleNet (Panis et al., 2016)	3.05
AGEn (Tan et al., 2017)	2.96
C3AE (Zhang et al., 2019)	2.95
ADPF (Wang et al., 2022)	2.86
Swin Transformer (Liu Z. et al., 2021)	2.69
Anet+Gnet+Rnet (Deng et al., 2021)	2.59
BridgeNet (Hou et al., 2016)	2.56
ABC+Swin Transformer (Ours)	**2.52**

Bold values represent the best data.

### 4.5. Evaluations on the Adience dataset

In this study, the images of the Adience dataset without age labels or confusing age labels in the dataset were removed. Experimentally, when using the Adience dataset for training and testing, the dataset was split into five groups using the 5-fold cross-validation method: 0-, 1-, 2-, 3-, and 4-fold. Each time when training and testing, one group of images was used for testing, the remaining four groups were combined into a training set for training, and the training-testing was done a total of five times. The final test set of the age group classification results was taken as the average of the five times results. The Adience dataset used an evaluation method for single-age classification accuracy. The accuracy for the aforementioned settings of the Adience dataset are tabulated in Table 3.

**TABLE 3:** Comparison of age estimates on the Adience dataset.

Models	Accuracy of a one-class classification (%)
SVM-dropout (Shen et al., 2019)	45.1 ± 2.6
CNN+over-sampling (Hou et al., 2016)	50.7 ± 5.1
R-SAAFc1 (Zhang et al., 2017b)	52.9
R-SAAFc2 (Zhang et al., 2017b)	53.5
Classification (Liu et al., 2018)	54.0 ± 6.3
Chained Net (Zhang et al., 2017a)	54.5
DEX w/o IMDB-WIKI Pretrain (Rothe et al., 2018)	55.6 ± 6.1
ABC+Swin Transformer (Ours)	**56.1**

Bold values represent the best data.

The accuracy of each age class of folds on the Adience dataset is shown as follows. Due to the random grouping in the images, the accuracy of each fold is different. Overall, the accuracy of each age class is essentially equal between different folds. This demonstrates that the model is stable in training. The accuracy of each age class for the aforementioned settings of the Adience dataset are tabulated in Table 4.

**TABLE 4:** Accuracy of each age class on the Adience dataset.

Fold	0–2	4–6	8–12	15–20	25–32	38–43	48–53	60–100
0	82.59	37.70	39.38	32.92	72.36	39.51	30.31	52.73
1	85.31	59.38	41.29	35.68	58.65	37.11	31.32	52.19
2	64.45	65.19	58.04	17.03	76.95	35.16	19.38	61.46
3	50.00	71.63	38.02	36.13	61.98	40.63	21.88	44.38
4	84.58	62.95	61.97	29.84	71.38	33.99	27.40	50.00
Average	73.39	59.37	47.74	30.20	68.26	37.28	26.06	52.15

### 4.6. Ablation study

We conducted ablation experiments to demonstrate the effectiveness of each component of the ABC+Swin Transformer. Specifically, we used the number of heads of attention probes in the multiheaded attention mechanism as a variable to demonstrate the necessity of each component. The MAE values for the aforementioned settings of the MORPH Album II dataset are tabulated in Table 5.

**TABLE 5:** MAE values for the attention-based convolution (ABC) framework on the MORPH Album II dataset for different numbers of attention probes.

Number of attention probes	0	1	2	4	8	16
ABC+Swin Transformer (Ours)	2.243	2.239	2.228	2.170	2.212	2.236

Table 5 shows that when the number of attention probes of ABC was zero, the MAE obtained by the ABC+Swin Transformer on the dataset was better than that obtained by the Swin Transformer, which demonstrated that the convolution performed an effective function in the network and enhanced the network’s ability to learn the age information of facial images. The MAE obtained using different numbers of attention probes showed that the optimum results were obtained when the number of probes was four. The reason for this was the presence of four important regions with rich age-specific information in the face. The more the number of probes, the more completely the age-specific regions could be extracted. As the number of probes increased, the intervals noticed by different probes might overlap, leading to redundancy in information extraction, which was not conducive to the information extraction of the network.

As shown in Table 6, the network was difficult to converge when λ_1_ was too small (KL_loss_ took a dominant role) and a big performance degradation occurred when λ_1_ was too large (the overlap loss took a dominant role). Within a long reasonable range, our proposed method performed stably. The number of heads of the multihead attention mechanism was set to four.

**TABLE 6:** MAE values for the attention-based convolution (ABC) framework on the MORPH II for different values of λ_*1*_.

Value of λ_*1*_	1	5	7.5	10	12.5	15	20
ABC+Swin Transformer (Ours)	2.37	2.25	2.19	2.17	2.20	2.27	2.38

## 5. Conclusion

In this study, we proposed the ABC framework to improve the performance of face-based age estimation tasks and combined ABC with the Swin Transformer to obtain better prediction performance. Our framework combined the shallow convolution and the multiheaded attention mechanism using the shifted window approach. The shallow convolution used several layers of a convolutional network with few convolutional kernels to condense the information, enhance the features of the image, and process the input to the same size for stitching with the information of the later attentional network computations and the Swin Transformer. The multiheaded attention mechanism allowed the network to learn and find regions that contained rich age-specific information, and display these regions. Finally, the age-rich regions obtained by the ABC framework were spliced with the image that was initially processed by the Swin Transformer along the channel dimension, and the stitched image tensor was carried out to the subsequent network of the Swin Transformer together to compute the final age prediction.

The significant age feature regions obtained by ABC were stitched with the original input images and then passed through the Swin Transformer for face-based age estimation, which made excellent use of the ability of the Swin Transformer to mine long-distance dependencies and parallel computation to learn more dependencies between important age features. The addition of the ABC framework well compensated the image local sensitivity and translation invariance of the Swin Transformer. The ABC framework spliced the important regions that contained rich age-specific information into the original image, which could fully mobilize the long-dependency of the Swin Transformer, that is, extracting stronger features by learning the dependency relationship between different features. As a result, the entire network could not only extract the important face-based age information regions but also further improve the prediction accuracy using the ability of the Swin Transformer to learn the interrelationship between features.

Based on the evaluation of several benchmark datasets, ABC significantly improved the prediction accuracy compared with several state-of-the-art methods. Future studies should investigate the design of custom estimators to further improve the performance, for example, by further augmenting the convolutional network.

## Data availability statement

The original contributions presented in this study are included in this article/supplementary material, further inquiries can be directed to the corresponding author.

## Ethics statement

Written informed consent was obtained from the individual(s), and minor(s)’ legal guardian/next of kin, for the publication of any potentially identifiable images or data included in this article.

## Author contributions

CS had full access to all the data in the study and was responsible for the integrity of the data and the accuracy of the data analysis. CS contributed to concept and design with input from KZ, SZ, YW, and LL and drafted the manuscript with input from all authors. CS, SZ, and KZ contributed to statistical analysis. CS and KZ contributed to funding acquisition, administrative, technical, or material support. All authors contributed to acquisition, analysis, or interpretation of data, critical revision of the manuscript for important intellectual content, and approved the submitted version.
